# Novel insights from 3D models: the pivotal role of physical symmetry in epithelial organization

**DOI:** 10.1038/srep15153

**Published:** 2015-10-16

**Authors:** Abhishek Kurup, Shreyas Ravindranath, Tim Tran, Mark Keating, Philippe Gascard, Lorenzo Valdevit, Thea D. Tlsty, Elliot L. Botvinick

**Affiliations:** 1 University of California Irvine, Department of Biomedical Engineering, Irvine, USA; 2 University of California San Francisco, Department of Pathology, San Francisco, USA; 3 University of California Irvine, Department of Mechanical and Aerospace Engineering, Irvine, USA; 4 University of California Irvine, Department of Surgery, Irvine, USA

## Abstract

3D tissue culture models are utilized to study breast cancer and other pathologies because they better capture the complexity of *in vivo* tissue architecture compared to 2D models. However, to mimic the *in vivo* environment, the mechanics and geometry of the ECM must also be considered. Here, we studied the mechanical environment created in two 3D models, the overlay protocol (OP) and embedded protocol (EP). Mammary epithelial acini features were compared using OP or EP under conditions known to alter acinus organization, i.e. collagen crosslinking and/or ErbB2 receptor activation. Finite element analysis and active microrheology demonstrated that OP creates a physically asymmetric environment with non-uniform mechanical stresses in radial and circumferential directions. Further contrasting with EP, acini in OP displayed cooperation between ErbB2 signalling and matrix crosslinking. These differences in acini phenotype observed between OP and EP highlight the functional impact of physical symmetry in 3D tissue culture models.

Over the past decade it has become apparent that the mechanical properties of the extracellular matrix (ECM) play important roles in breast cancer^1^,^2^,^3^. One way in which cells can gauge these properties is through transmembrane receptors such as integrins^4^, which are activated by mechanical tension leading to downstream molecular signalling in a process called mechanotransduction^5^. Changes in integrin signalling and expression can drive epithelial to mesenchymal transition^6^, regulate cell-adhesion and migration^7^, and promote tumour progression^8^. Furthermore, blocking integrin activity with an exogenous ligand has been shown to reverse the malignant phenotype in mammary epithelial cells (MECs) *in vitro*^9^. Just as cells mechanically interact with the ECM via integrins, they interact with neighbouring cells via specialized protein scaffolds including cadherins, which play a key role in cell-cell adhesion and force transmission^10^. Cadherins also play a prominent role in maintaining MEC polarization and homeostasis^11^. Similar to integrins, cadherin-mediated force transduction correlates with substrate rigidity and has been implicated in cancer^10^,^12^,^13^.

In order to better understand both biochemical and mechanical cell-ECM and cell-cell interactions in a physiologically relevant context, 2D cell culture has been largely replaced with 3D cell culture^14^. While 3D tissue culture models are no substitute for animal models, they do allow engineering control over system architecture, molecular transport, mechanical stresses, growth factors, and other aspects found *in vivo* and, in that regard, are superior to 2D culture^15^,^16^,^17^. 3D culture methods are also compatible with multi-well plate arrays and lab-on-a-chip formats for use in high-throughput screening studies^16^,^18^. For example, it has been shown that 3D hepatocyte culture was superior to 2D in drug toxicity testing and recapitulated the results found *in vivo*^19^.

3D cell culture is commonly used in breast cancer biology. In these studies MECs are cultured within or on a hydrogel containing laminin-rich reconstituted basement membrane (rBM), such as Matrigel^14^. MECs cultured with rBM form 3D, hollow, growth-arrested and polarized acini that resemble the glandular milk producing lobules of the breast *in vivo*^20^. Importantly, they do not form these structures in 2D^21^. Relating these studies to physiological relevance, the processes of acinus disruption within *in vitro* 3D models have been shown to be similar to those observed *in vivo*^21^. During acinus disruption, *in vitro* growth-arrested acini exhibit initiation of proliferation, apoptosis evasion, and polarization loss^22^. Downstream of acinus disruption, upon progression towards metastasis, cancerous cells invade the nearby matrix via increased matrix metalloproteinase (MMP)-mediated remodelling, cell-generated forces and migration^9^,^22^,^23^.

There are two predominant 3D culture methods in the field of breast cancer: the overlay protocol (OP) and the embedded protocol (EP) (Fig. 1a). What is not yet clear is which of these two methods of 3D cell culture is the most appropriate for investigating the effects of the ECM on MEC acini phenotype. Indeed, these methods differ in the way acini interact with the ECM. In OP, the more commonly used of the two, cell colonies are cultured atop a thin film of ECM, typically comprised of Matrigel and extracellular molecules such as type 1 collagen. The culture is supplemented with overlay medium (OM) consisting of assay medium plus 2–4% Matrigel (see methods). Matrigel in the OM forms a thin membrane around the otherwise free surfaces of the acini such that cells are in a 3D ECM environment (Fig. 1a, *top*)^20^. On the other hand, in EP, cells are completely submerged within the ECM (Fig. 1a, *bottom*). While acini form in both conditions, EP is used infrequently in the literature compared to OP because the OM can be easily washed off allowing direct access to the cells for staining, imaging, and harvesting^20^.

Experimentally, acini are slightly submerged within the ECM in OP. This configuration is an important aspect of OP because the interface between the ECM bed and OM introduces a discontinuity in the mechanical environment. Therefore, when investigating ECM resistance to deformation under cell contractile forces one cannot consider the mechanical properties of the ECM alone, but must also consider the geometry of the system. In OP, the fluid surface of the laminin-rich OM creates a 3D biochemical, but a 2D mechanical microenvironment. In this study, we sought to determine whether the physical natures of OP and EP could influence MEC acini homeostasis and disruption. We find that physical symmetry is dominant over ErbB2 signalling and/or matrix crosslinking in influencing acinar phenotype.

## Results

### Significant differences between OP and EP for cell-ECM and cell-cell mechanical stresses

**Figure 1:**
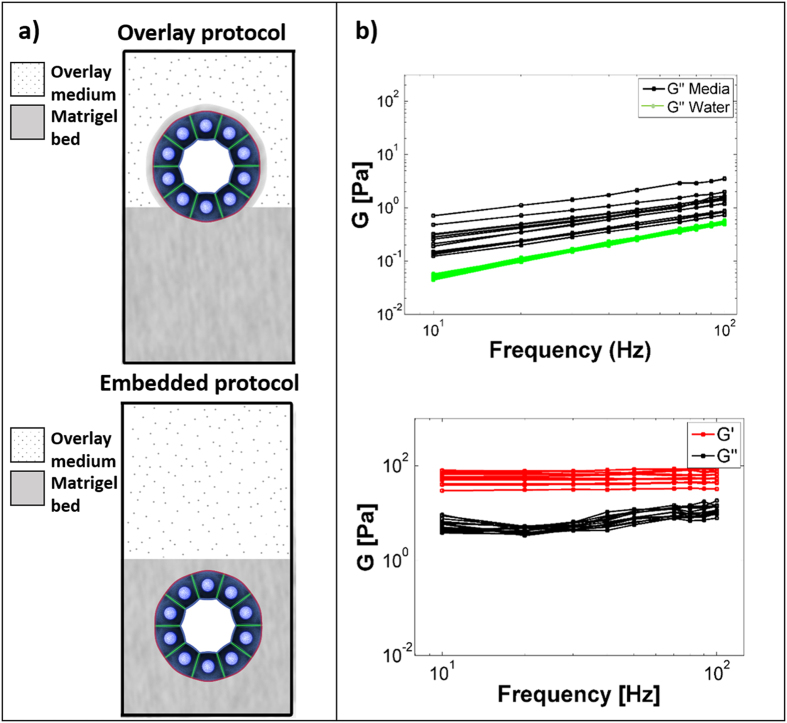
Mechanical properties of EP and OP. (**a**) Schematic representation of the overlay and embedded culture methods. Red corresponds to β4-integrin, green to cadherin, and light blue circles are representative of the nuclei. (**b**) Frequency spectra for the complex shear modulus G of water, OM and the Matrigel bed as determined by AMR. G’ and G” frequency sweeps of 10, 20, 30, 40, 50, 70, 80, 90, and 100 Hz are shown. Each line represents a different bead. 10, 13, and 13 beads were measured for water, OM and Matrigel, respectively. Statistical significance was determined with one-sided Mann-Whitney U tests with p-values < 0.025 deemed significant.

Cell-cell and cell-ECM mechanical stresses depend on the resistance of the ECM to deformation, which in turn depends on both material properties and the geometry of the system. To understand stresses acting on and within an acinus cultured according to either OP or EP, we first used optical tweezers active microrheology (AMR)^24^ to measure the viscoelastic shear modulus (*G*, a material property) of OM and Matrigel. *G* is a complex parameter with *G* = *G’* + *iG”*, where *G’* and *G”* are the shear storage (elastic) and loss moduli (viscous), respectively. We first investigated whether the addition of Matrigel to culture medium (to form OM) would transform the purely viscous medium into a material capable of storing elastic energy that could react to cell contractile forces and sustain stresses acting through cell-ECM adhesions. Our results showed that G’ was equal to zero for OM, indicating that OM could not store elastic energy. Furthermore, G” was linearly dependent on frequency, indicating that OM behaved as a viscous fluid (Fig. 1b, *top*)^25^. In contrast, Matrigel exhibited a *G’* ~ 60 Pa (Fig. 1b, *bottom*), confirming that, unlike OM, Matrigel could store elastic energy. Values for *G”* across tested frequencies of oscillations were insensitive to frequency, which is typical for hydrogel systems. These results demonstrated that the OM could not resist static tensile stresses, whereas Matrigel (conditions found in EP) could.

**Figure 2:**
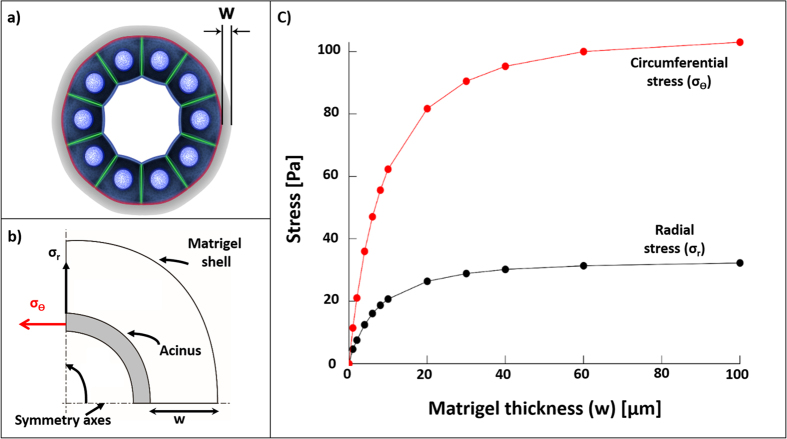
Finite Element Analysis of adhesive and cell-cell stresses as a function of Matrigel thickness. (**a**) An acinus surrounded by Matrigel with thickness *w*. (**b**) Schematic of the acinus quadrant analysed by FEA with outer radial (*σ*_*r*_) and midpoint circumferential (*σ*_*θ*_) stresses. *σ*_*r*_ represents adhesive stresses between acinus and surrounding Matrigel. *σ*_*θ*_ represents stresses acting with the wall of the acinus along cell-cell adhesions. (**c**) Computed stresses with 10% contraction of acinus when *w* is increased from 0–100 μm.

In the first of two models, we examined the effect of the volume of Matrigel surrounding an acinus on cell-cell and cell-ECM stresses by formulating and solving models by Finite Element Analysis (FEA) in ABAQUS (Dassault Systemes). The acinus was modelled as a thick-walled spherical shell having a defined internal diameter *D* = 100 μm and wall thickness *t* = 10 μm. The acinus was surrounded by a spherical shell of Matrigel with thickness (*w*) ranging from 1 to 100 μm (Fig. 2a). Our model imposes a 10% uniform contraction of the acinus and computes the resulting normal stresses in the radial and circumferential directions. In the figure, the radial stress (σ_r_) is probed at the outer surface of the acinus and acts in the radial direction, and the circumferential stress (σ_θ_) is probed at the midpoint of a cross-section of the acinus wall and acts in the circumferential direction (Fig. 2b). σ_r_ and σ_θ_ are representative of cell-ECM adhesion and cell-cell junction stresses, respectively.

Figure 2c plots values of σ_r_ and σ_θ_ with increasing Matrigel shell thickness, *w*. Both σ_r_ and σ_θ_ are asymptotic at large values of *w*, where stresses are no longer sensitive to changing values of *w*. In other words, at large values of *w* (Matrigel thickness), OP system converges to EP model. Thus, this simulation compares stresses between OP and EP. Of particular note, when *w* is ~1 μm (typical for OP), σ_r_ and σ_θ_ are one order of magnitude weaker than their asymptotic values. Thus, when viscoelastic properties of the Matrigel are held constant, the thickness of the Matrigel shell alone can modulate stresses to a large degree, highlighting an important difference between OP and EP.

**Figure 3:**
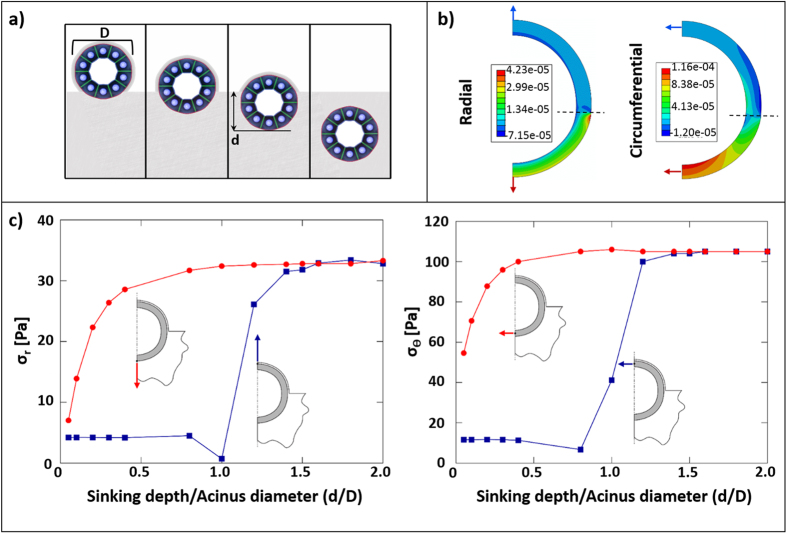
Finite Element Analysis of OP, where the free surface of an acinus is coated by a 1 μm thick film of Matrigel. The bottom surface of the acinus is resting on or submerged (partially or completely) within Matrigel. (**a**) An acinus of diameter *D* was modelled with varying sinking depths *d* inside the Matrigel bed. Outer radial (*σ*_*r*_) and midpoint circumferential (*σ*_*θ*_) stresses following a 10% contraction were computed for a half circle. (**b**) Colour maps of stresses for (*d/D* = 0.4). Hashed lines indicate the Matrigel-OM boundary. Units are in megapascals. (**c**) *σ*_*r*_ and *σ*_*θ*_ are shown as a function of sinking depth (*D/d*). Red lines (and arrows) correspond to the bottom of the acinus and blue lines (and arrows) to the top.

In the second model, we included the ECM bed on which an acinus would form in OP. We computed σ_r_ and σ_θ_, for an acinus partially submerged within the Matrigel bed with the free surface coated by a 1 μm-thick shell of Matrigel, as found in OP (Fig. 3a). The depth of submersion, *d*, was varied between zero (acinus just touching the Matrigel bed) and 2 × *D* (acinus deeply submerged, as in the case for EP). As shown in Fig. 3b, stresses changed with respect to both the radial and circumferential coordinates. Values of σ_r_ and σ_θ_ at the top and bottom surfaces of the acinus as a function of increasing values of *d/D* are plotted in Fig. 3c. At large values of *d/D*, σ_r_ and σ_θ_ at both surfaces increased asymptotically. Asymptotic values of stress are representative of EP and in agreement with the first model (Fig. 2c). For intermediate values of *d/D*, both σ_r_ and σ_θ_ increased with *d/D* at the bottom surface of the acinus. In contrast, stresses at the top surface were insensitive to *d/D* until *d/D* = 1, i.e. when the top surface of the acinus first entered the bed. For *d/*D = 0.4, σ_r_ and σ_θ_ were an order of magnitude larger at the bottom surface as compared to the top.

Taken together, these two analyses indicated that stresses acting on cells in an acinus are significantly different between OP and EP both in terms of stress magnitude as well as distribution. Consequently, cell-cell and cell-ECM mechanical stresses are in fact different between OP and EP, which may result in differences in acinus morphology and phenotype.

### Dimensionality is as potent as cross-linking and oncogenic activation in determining phenotype

To test further for the effects of culture dimensionality on cell biology, we cultured MECs in OP and EP under three experimental conditions previously shown to promote hyperplastic or invasive phenotypes^22^,^26^. In our experiments, the OM was as described above and the bed comprised of a 1:1 mixture of Matrigel and type I collagen. The first experimental condition was type 1 collagen crosslinking, a condition chosen because covalent collagen crosslinking of the stroma mediated by lysyl-oxidase (LOX) has been found to promote tumour progression in mice^27^. Clinically, breast cancer patients with ER-negative tumours and overexpression of LOX have poor survival^27^. *In vitro*, non-covalent crosslinking by collagen glycation (i.e. ribose-mediated^28^) was used to test the impact of stromal crosslinking on MEC acini structure and invasiveness^29^. The second experimental condition relied on activation of ectopic avian erythroblastosis oncogene B (ErbB2), a proto-oncogene up-regulated in 25% of metastatic breast cancers and 20–80% of Ductal Carcinoma *in Situ* (DCIS)^22^,^30^. As a model of ErbB2 activation, we cultured MCF10A.ErbB2 cells, a line developed by the Muthuswamy laboratory. This cell line expresses a chimaeric ErbB2 receptor which can be dimerized and activated upon addition of an exogenous synthetic ligand^22^. The third experimental condition was a combination of collagen crosslinking and ErbB2 activation, a condition that has been shown to promote acini invasion in OP using the same cell lines and ECM as those described in our experiments^26^.

MCF10A.ErbB2 were cultured in OP or EP within 12-well plates for 14 days. On day 15 the medium was substituted to generate three experimental conditions: *(1) Ribose*: ribose-mediated collagen crosslinking, *(2) ErbB2*: ErbB2 signalling activation, and *(3) ErbB2*+*Ribose*: both ErbB2 activation and collagen crosslinking. Cells were cultured in each experimental condition from days 15 to 30. On day 30, cells were fixed in formalin and imaged to assess acinus polarization (after staining for β4-integrin), lumen filling (after DAPI staining), and morphology (by fluorescence laser scanning confocal microscopy and transmitted light imaging). Four acinus categories were defined as summarized in Table 1.

#### Acini in OP are larger and less round as compared to acini in EP

**Figure 4:**
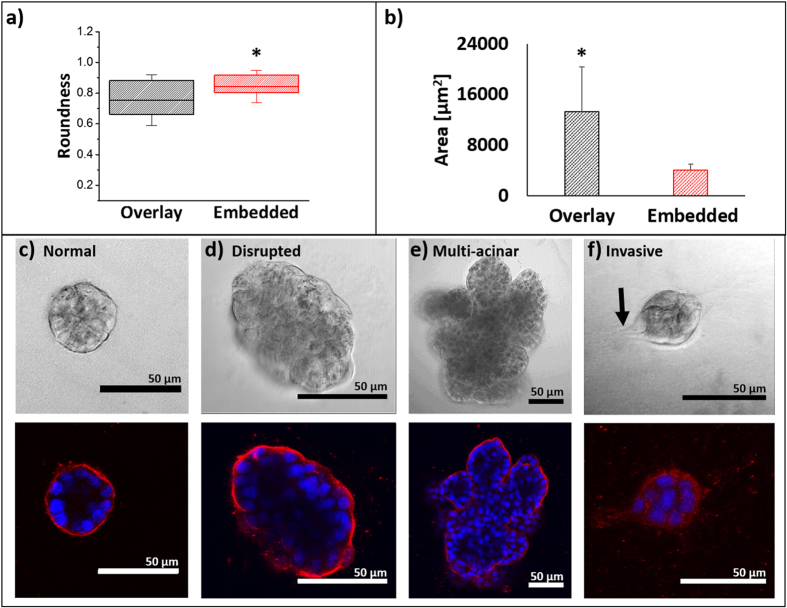
Acinus morphology in EP and OP. Roundness (**a**) and area (**b**) of acini in EP and OP. Acini were classified from images according to 4 categories: Normal (**c**), Disrupted (**d**), Multi-acinar (**e**), and Invasive (**f**). Representative transmitted light *(top row)* and confocal fluorescence *(bottom row)* images are shown; β4-integrin immunocytochemical staining (red) and nuclear staining of DAPI (blue). Black arrow in (**f**) denotes an invasive protrusion. Scale bars are 50 μm. Samples sizes for OP and EP: 791 and 1141 acini, respectively. Statistical significance was assessed using a one sided Mann-Whitney U tests. p-values < 0.05 are noted with an asterisk.

In total, transmitted light images for over 1900 colonies were analysed by processing via algorithms developed with MATLAB (Mathworks) to calculate the cross-sectional area and roundness of each acinus and to categorize each acinus according to Table 1. For each image, the boundary of an acinus, imaged at its midsection, was manually traced. We defined *perimeter* as the length of the trace, *area* as the area within the traced region, and *roundness* as 4 x π x *area*/(*perimeter*^2^). Acini in EP were found to be more round (Fig. 4a) and smaller (Fig. 4b) than in OP. Furthermore, acini in EP exhibited less variability in *roundness* and *area* than in OP, consistent with previous reports of smaller and more uniform acini in EP as compared to OP^20^. Our results are not surprising from a mechanical perspective because EP offers a symmetrical physical environment with greater resistance to expansion, which is predicted to limit acinus growth and to promote a more spherical phenotype.

#### Matrix crosslinking coupled with ErbB2 activation in OP promotes invasion and growth

**Figure 5:**
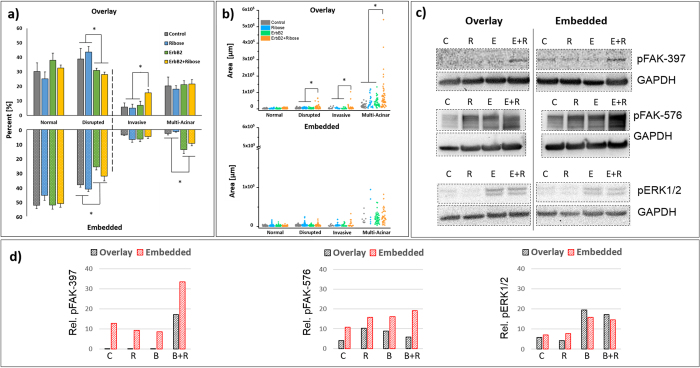
Acinus phenotype for *control*, *ribose*, *ErbB2* and *ErbB2*+*ribose* conditions. (**a**) Percentage of acini adopting *normal*, *disrupted*, *invasive*, and *multi-acinar* morphologies for OP (*top*) and EP (*bottom*). The vertical dashed line separates similar (left of the line) and different (right of the line) outcomes between the two protocols. (**b**) Area of acini in the four categories for OP (*top*) and EP (*bottom*). Horizontal bars indicate grouping of conditions for statistical testing. For percentage of acini, differences were considered significant if p-values were  < 0.0125 to account for multiple comparisons using a one-sided Student’s t-test. For area, differences were considered significant for p-values < 0.05 using a one-sided Mann-Whitney U test. Sample size for EP and OP: 1141 acini and 791 acini, respectively, counted across 3 repeat experiments. (**c**) Western blot analysis of lysates from MCF10A.ErbB2 cells grown in OP or EP under *control* [C], *ribose* [R], *ErbB2* [E], and *ErbB2*+*ribose* [E+R] conditions. pFAK-397 (top row), pFAK-576 (middle row), and pERK1/2 (bottom row). Extracts were probed for GAPDH to normalize sample input. p-ERK 1/2 was probed using the streptavidin-biotin sandwich method and the other two with secondary antibodies. Chemiluminescence was used to detect all bands. Experimental conditions were the same across all samples. Blots were cropped for clarity of presentation (hashed boxes). Full-length blots are presented in Supplementary Fig. 1. (**d**) Relative expression of each phosphorylated protein normalized to GAPDH from (**c**). At least two blots were analysed to assess the phosphorylation status of each protein. (**c**,**d**) are representative of one of the data sets.

The percentage of *normal, disrupted, invasive and multi-acinar* colonies cultured in OP are shown in Fig. 5a *(top)*. The percentage of *normal* acini was independent of culture condition (*control, ribose, ErbB2*, and *ErbB2*+*ribose*). In contrast, the percentage of *disrupted* colonies decreased significantly (p < 0.01) with ErbB2 activation. Additionally, the percentage of *invasive* colonies was greater for *ErbB2*+*ribose* as compared to the other conditions, a finding consistent with published work^26^ that supports cooperation between the ErbB2 and collagen crosslinking-mediated mechanosensing pathways. Surprisingly, the percentage of *multi-acinar* structures was not dependent on culture condition, an unexpected result considering that two of these culture conditions were without dimerizer. Consistent with our finding, it has been shown that MCF10A.ErB2 cultured in OP without dimerizer spontaneously form *multi-acinar* structures^31^. However, the relative incidence of these structures as a function of the presence or absence of dimerizer has not been previously reported.

The values for the area of *normal* acini were independent of culture condition and homogeneous while the values for the areas of *disrupted*, *invasive* and *multi-acinar* colonies were significantly greater and more heterogeneous for *ErbB2*+*ribose* compared to any other culture condition (Fig. 5b, *top*). Larger acini attested to more proliferation in the *ErbB2*+*ribose* conditions, further supporting the theory of cooperation between matrix crosslinking and ErbB2 activation in driving tumour growth.

#### Matrix crosslinking coupled with ErbB2 activation in EP does not promote invasion or growth

As in OP, the percentage of *normal* acini in EP was independent of culture condition and the percentage of *disrupted* acini decreased with ErbB2 activation (Fig. 5a*, bottom*). Interestingly, we observed two striking differences in acinus phenotype between EP and OP. First, in contrast with OP, the percentage of *invasive* colonies in EP under the *ErbB2*+*ribose* condition did not increase as compared to the three other conditions. Second, the percentage of *multi-acinar* colonies increased significantly with ErbB2 activation as compared to those conditions without ErbB2, only in EP. Additionally, the percentage of *multi-acinar* colonies in the control condition was lower for EP than OP, as previously observed in a study of colonies of MCF10AT cells with activated H-Ras^32^. We speculate that the lower percentage of *multi-acinar* colonies in EP is due, in part, to increased mechanical stresses (relative to colonies grown in OP) resulting from physical confinement within the ECM, as supported by our FEA models. As seen with OP, the area of *normal* acini in EP was independent of culture condition (Fig. 5b, *bottom)*. However, in contrast to OP, the areas of *disrupted*, *invasive*, and *multi-acinar* colonies were not significantly greater for *ErbB2*+*ribose* compared to the other culture conditions combined (compare Fig. 5a, *bottom* to Fig. 5b, *top*). The differential sensitivity to collagen crosslinking and ErbB2 activation for OP and EP indicates a significant role of the physical asymmetry in determining acinus phenotype, where that role may be more potent than the chemical perturbations.

#### FAK but not ERK signalling differs between OP and EP

The observations above led us to predict that differences in mechanotransduction-related signalling would be observed between OP and EP. To investigate whether such differences would occur across experimental conditions we monitored expression levels of activated states of key effectors in canonical signalling pathways (Fig. 5c,d). First, we assayed Focal Adhesion Kinase phosphorylated at Y397 (pFAK-397) and Y576 (pFAK-576) as read-outs of stretch-mediated integrin clustering during the formation of focal contacts, a hallmark of mechanotransduction^33^. FAK is recruited to focal contacts through interaction with the cytoplasmic tails of β1, β3 and β5-integrins^33^. Once recruited, FAK clusters and is autophosphorylated at Y397^34^. Thus, pFAK-397 is a good indicator of adhesion-mediated mechanotransduction. In OP, pFAK-397 levels were too low to be detected in *control, ribose*, or *ErbB2* conditions, but was detectable in the *ErbB2*+*ribose* condition (Fig. 5c, *top*). In contrast, pFAK-397 levels were detected for all four conditions in EP, with the strongest level found in the *ErbB2*+*ribose* condition (Fig. 5d, *left*). pFAK-397 has increased affinity to the SH2 domain of Src, promoting binding to Src, which in turn mediates several additional tyrosine phosphorylation events on FAK, including phosphorylation at Y576 and Y577^34^. Phosphorylation of FAK at these tyrosine residues is actually required to transform the FAK-Src complex into its highest signalling state^35^. Therefore phosphorylation of FAK at Y576 and Y577 is indicative of the full engagement of downstream mechanotransduction-dependent signalling. Similar to our observations with pFAK-397, pFAK-576 levels were greater in EP across all conditions as compared to OP (Fig. 5d, *middle*). Taken together, these differences in phosphorylation events of FAK support increased adhesion-mediated mechanotransduction in EP as predicted by our model.

We also examined the activation of extracellular regulated signalling kinase 1 and 2 (ERK1/2), which is known to regulate cell proliferation, cell migration^1^ and acinus disruption^36^. ERK1/2 is also necessary for the onset of epithelial to mesenchymal transition and invasion in cancer^37^. Levels of pERK1/2, the activated state of ERK1/2, were not different between OP and EP across all conditions (Fig. 5c, *bottom*), which was surprising considering differences in colony size (Fig. 4b). Nevertheless, pERK1/2 levels were low in *control* and *ribose* conditions as compared to *ErbB2* and *ErbB2*+*Ribose* conditions in both OP and EP (Fig. 5d, *right*). Matrix crosslinking seemed to play a minor role since acini grown in the *ErbB2* condition already showed maximal pERK1/2 phosphorylation. Together, these data supported a predominant role for growth-factors in engagement of ERK signaling. In line with our finding, Raghavan *et al*. have reported that ERK activation in Madin-Darby canine kidney epithelial cells-derived acini does not depend on culture condition dimensionality (monolayer culture or 3D) but rather on the addition of exogenous growth factors^11^,^38^.

## Discussion

Here we investigated OP and EP for any protocol-dependent effect on acini formation that may impact mechanical hypotheses testing. While it has previously been speculated that OP may result in altered phenotypes as compared to EP^9^, our study provides direct evidence for phenotypic differences due to the geometry of the culture system. A novel insight is provided by our data where three independent variables (ECM crosslinking, ErbB2 activation and ECM dimensionality) were tested. Strikingly, our data suggest that ECM dimensionality is the dominant determinant of acini phenotype in our experiments. This result is exemplified by the observations that the percentages of *multi-acinar* colonies were independent of culture conditions only in OP and that cooperation between ErbB2 signalling and matrix crosslinking to drive invasion was lost when switching from OP to EP.

The observed phenotypical differences are interesting considering the important roles played by integrins and cadherins in establishing and maintaining MEC polarization and homeostasis^9^. These two molecular families contribute to protein complexes known to transduce forces acting along radial and circumferential axes, respectively. Therefore, cells grown according to OP or EP would be expected to develop into different phenotypes. This expectation follows our finite element analysis which shows that the circumferential and radial forces required to maintain acinar architecture are very different between OP and EP, even if the material properties of the ECM are identical. In particular, the thin coating of ECM in OP is easily deformed by the contracting acinus, thus maintenance of acinar shape requires small magnitude mechanical stresses as compared to EP. Consequently, stresses acting through integrins and cadherins are dissimilar for otherwise identical OP and EP experiments, and likely lead to differential signalling. This finding is consistent with our observations of differences in integrin-mediated signalling between OP and EP. Indeed, colonies in OP exhibited lower levels of pFAK-397 and pFAK-576 than in EP, indicating that the potential of the ECM to store elastic energy as well as ECM physical symmetry are predominant determinants of force-mediated integrin signalling.

In addition to integrin-mediated signaling, we examined levels of phosphorylated ERK (pERK), since ERK activation is often associated with tumorigenesis and cancer progression in humans^39^. Our results support that, in our experimental conditions, adhesion signalling alone is a weak activator of ERK1/2, while ErbB2 signaling remained a potent activator as expected. It has been previously shown that p-ERK levels increase with ErbB2 activation in MCF10A.ErbB2 cells^40^, as observed here in both OP and EP across all conditions. Because ERK activation plays a key role in promoting proliferation, we expected the percentage of multi-acinar colonies (Fig. 5a) to reflect higher p-ERK1/2 levels (Fig. 5d). While this correlation was confirmed in EP, in contrast, the percentage of *multi-acinar* colonies in OP was insensitive to ERK1/2 activation and consistently showed high percentages of* multi-acinar* colonies as compared to EP control.

Of particular note, we also monitored the activation of AKT, which acts downstream of the PI-3 kinase (PI3K) pathway to enhance cell survival^41^. Surprisingly, levels of pAKT, the phosphorylated state of AKT, could not be detected by western blot analysis in any of the conditions tested (our unpublished data). It has been extensively documented that the PI3K/AKT pathway is often used by cancer cells as a back-up to drive tumour progression when the MAPK/ERK pathway is no longer active or is blocked with therapeutics^42^,^43^.

In conclusion, even though OP has been used to study mechanics in tumour progression using various ECMs^2^,^4^,^9^,^22^,^44^,^45^, EP is more appropriate when considering a model more representative of the relatively homogeneous mechanical environment of the breast. While it is granted that neither method fully recapitulates physiological conditions, the geometry of EP better captures the 3D physical environment in comparison to OP and therefore should be employed when investigating matrix mechanics under uniform mechanical conditions. On the other hand, OP is a superior choice when investigating matrix asymmetry. It is well documented that whereas stiffness is relatively homogeneous in disease-free breast tissue or breast tissue with benign lesions, one can observe steep gradients of stiffness within a tumour^46^,^47^,^48^. In that respect, characterizing the physical differences between an embedded model and an overlay model is an important component of our understanding of disease progression using 3D culture models.

## Methods

### Cell Culture

MCF10A.ErbB2 cells were cultured in 3D conditions with appropriate media formulations^20^. Briefly, cells were first cultured in plastic dishes with Growth medium (5% horse serum, 0.5 μg/ml hydrocortisone, 20 ng/ml human epidermal growth factor (hEGF), 10 μg/ml Insulin, 100 ng/ml cholera toxin, and 100 units of Penicillin/Streptomycin in DMEM/F-12 media). Cells were trypsinized at 70% confluency for 3D culture experiments using a 50/50 Matrigel/Collagen ECM (BD Biosciences). The final collagen concentration was 1.6 mg/ml. Assay medium (same as Growth medium except 2% instead of 5% horse serum) was mixed with 5 ng/ml HEGF and 2% Matrigel to make OM^9^. For EP, 300 μl of cell suspension (at 70,000 cells/ml ECM) per well was plated in 12-well glass bottom plates (*In vitro* Scientific) and fed with OM. For OP, 2 ml of OM was mixed with 20,000 cells and plated in each well on top of 200 μl of pre-polymerized ECM.

ECM was gelled for 45 minutes in a standard 37 °C humidified cell culture incubator with a 5% CO_2_ environment. Both EP and OP cell cultures were fed every four days with fresh OM. For the ErbB2 condition, HEGF in assay medium was replaced with 1 μM dimerizing ligand (B/B Homodimerizer, Clontech). For the ribose condition, 15 mM Ribose (Sigma) was added to the assay medium. In the ErbB2+Ribose condition, HEGF was replaced with B/B Homodimerizer and ribose was added to the assay medium. Both dimerizing and ribose media were added starting at week 3 to appropriate dishes with media changes every four days.

Immunocytochemical staining of β4-integrin was conducted as previously described^20^ using a primary mouse anti-human β4-integrin antibody (Millipore) and a secondary goat anti-mouse antibody coupled to Alexa 488 (Life Technologies). Nuclei were stained with DAPI (Life Technologies). Blocking and antibody incubation were all conducted overnight for EP. Western blots were conducted as described in supplementary materials.

### Active Microrheology

Optical tweezers active microrheology (AMR) was performed as previously described^24^. Briefly, 2 μm diameter carboxylated beads (Bangs Laboratory) were trapped within a focused 1064 nm laser microbeam (IPG Photonics) and oscillated at different frequencies. A 785 nm (World Star Tech) non-steered detection microbeam is deflected by the movement of the bead and corresponds to bead position in time, which is detected by a position sensitive quadrant photodiode. A complex shear modulus can be determined by analysing the phase-amplitude relationships between the trapping laser and the detection laser positions.

Beads were either embedded within 200 μL of Matrigel or mixed with OM (2.2 ml assay medium + 2% Matrigel) in a 35 mm glass bottom dish. 2 ml of assay medium was added to Matrigel to keep it hydrated. 10–13 beads were measured by AMR at frequencies of 10, 20, 30, 40, 50, 70, 80, 90 and 100 Hz. A custom-built on-stage incubator was used to maintain a temperature of 34 °C.

### Finite Element Analysis

Numerical estimates of cell-cell and cell-ECM stresses were obtained by Finite Elements analysis. All simulations were performed with the commercial software ABAQUS Standard (Dassault Systemes). The acinus was modelled as a thick spherical shell, with internal diameter *D* = 100 μm and thickness *t* = 10 μm. In the first set of simulations, an acinus was embedded in a spherical Matrigel shell of thickness ranging from 1 to 100 μm. In the second set of simulations, an acinus was coated with a 1 μm-thick Matrigel layer and partially submerged in a deep Matrigel bed. The penetration depth, *d*, ranged between 0 (acinus just touching the Matrigel bed) and 2 × *D* (acinus deeply submerged in Matrigel bed).

2D axisymmetric quadratic elements were used for both the acinus and the Matrigel, in all simulations. That is, only one quadrant of the circular domain was modelled for EP simulations, with symmetry boundary conditions applied to the symmetry axis (Fig. 2b). For OP simulations, the bottom side of the block was allowed to deform horizontally, but not vertically. No pressure was allowed to build up during deformation for either simulations since tight junctions of an acinus have been shown to be leaky and allow for transport of interstitial fluid (with the exception of the lactation phase, when a positive pressure can be maintained within the lumen^49^,^50^). Both the acinus and the ECM are modelled as linear elastic materials, with a Young’s modulus of 720 Pa and 450 Pa, respectively. These values were based on AFM measurements reported in the literature for MCF10A cells^51^ and Matrigel^52^. A Poisson’s ratio of 0.5 was used for both materials, to simulate a soft incompressible solid.

Contraction of the acinus was simulated by imposing a fictitious coefficient of thermal expansion to the acinus only and prescribing a temperature drop, resulting in a tendency to contract by 10% in all directions. This free contraction is resisted by the constraining effect of the Matrigel, albeit by different amounts in OP and EP simulations. In both cases, a stress field develops in the acinus and Matrigel. Radial and circumferential stresses in the acinus at different locations were extracted and plotted.

### Phenotype Classification

Described in supplementary materials.

### Statistical Analysis

Experiments were conducted in triplicates with approximately 60–100 acini counted for each condition. One-tailed Student’s t-test and Mann-Whitney U tests were performed in OriginPro to determine statistical significance. The alpha value to determine statistical significance in acini percentages was chosen using the Bonferroni correction (see supplement).

## Additional Information

**How to cite this article**: Kurup, A. *et al*. Novel insights from 3D models: the pivotal role of physical symmetry in epithelial organization. *Sci. Rep*. **5**, 15153; doi: 10.1038/srep15153 (2015).

## Supplementary Material

Supplementary Information

## Figures and Tables

**Table 1:** Acinus categories and their corresponding description.

Category	Description			
	Morphology	Polarization(β4-Integrin)	Lumen(DAPI)	Shape
Normal (Fig. 4c)	Round	Yes	Yes	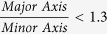
Disrupted (Fig. 4d)	Oval, irregular shape, bulgy	Partial	Partial	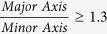
Multi-Acinar (Fig. 4e)	Berry-shaped; multiple lobules	Partial	Partial	
Invasive (Fig. 4f)	Protrusion(s). General loss of spherical geometry	Loss	Loss	

Confocal fluorescence imaging was used to create the categories based on β4-integrin polarization and the presence of a hollow lumen, as determined by immunocytochemistry and DAPI staining, respectively. Morphology was assessed manually from transmitted light images. A shape threshold of 1.3 was determined empirically.
