# Supplementary material for: Novel insights from 3D models: the pivotal role of physical symmetry in epithelial organization
Source: Sci Rep. 2015 Oct 16;5:15153. doi: 10.1038/srep15153 (PMC4608012; doi:10.1038/srep15153)
Supplement: Supplementary Information [file srep15153-s1.pdf]

Novel insights from 3D models: the pivotal role of physical symmetry in epithelial organization

Abhishek Kurup[1], Shreyas Ravindranath[1], Tim Tran[1], Mark Keating[1], Philippe Gascard[2], Lorenzo Valdevit[3], Thea D. Tlsty[2], Elliot Botvinick[1, 4,*]

[1] University of California Irvine, Department of Biomedical Engineering, Irvine, USA

[2] University of California San Francisco, Department of Pathology, San Francisco, USA

[3] University of California Irvine, Department of Mechanical and Aerospace Engineering, Irvine, USA

[4] University of California Irvine, Department of Surgery, Irvine, USA

*ebotvini@uci.edu

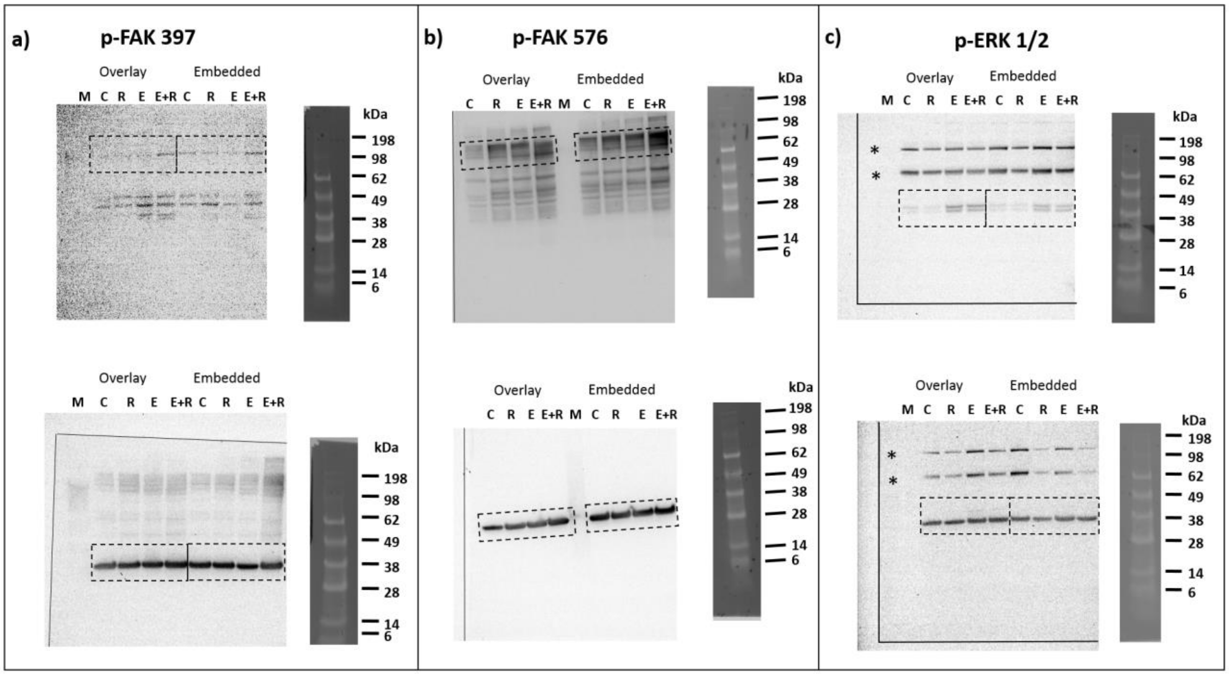

Supplementary Figure 1: Western blot analysis of lysates of control [C], ribose [R], ErbB2 [E], and ErbB2 + ribose [E+R] MCF10A.ErbB2 cells grown in OP or EP conditions. Cell lysates were probed for: (a) p-FAK 397, (b) p-FAK 576, and (c) p-ERK 1/2 (*top panels*). Blots were subsequently stripped and probed for GAPDH to normalize for sample input (*bottom panels*). Extra bands in (a, *bottom panel*) resulted from prior probing for phosphorylated tyrosine kinase (data not shown) and incomplete stripping. [M]: molecular weight marker lane. Corresponding molecular weights are shown on the right side of each panel. *: non-specific bands resulting from streptavidin-HRP interactions. Hashed boxes: cropping used to generate the panels shown in **Figure 5d**.

Western blot analysis

The protein extraction protocol was adapted from a previously described method[1]. Briefly, after four weeks of 3D culture as described in the methods, culture medium was aspirated from wells and cells were treated with pre-warmed Collagenase Release Solution, consisting of DMEM: F12 (Life Technologies) supplemented with 0.05%Trypsin (Life Technologies), 1% FBS, 200 U/ml Collagenase IV (Life Technologies) and 50 U/ml Dispase (Corning) for 15 minutes at 37°C followed by rigorous pipetting and further incubation for 15 minutes at 37°C. Pipetting and incubation cycle was repeated 3-5 times until a uniform slurry was obtained. The slurry was centrifuged, the supernatant discarded and the pellet resuspended in ice cold PBS containing a cocktail of protease and phosphatase inhibitors (cOmplete ULTRA and phosSTOP tablets, Roche) and centrifuged for 5 minutes at 4°C. This process was repeated twice. The pellet was resuspended in freshly prepared lysis buffer (50 mM Tris-HCl, pH 7.5 (Life Technologies), 150 mM NaCl, 1% v/v IGEPAL CA-630 (Sigma), 5mM EDTA (Life Technologies), and cocktails of protease and phosphatase inhibitors (described above) and incubated on ice for 30 minutes followed by rapid trituration using a 25G x 5/8 syringe needle (Sigma). The obtained cell lysates were centrifuged. The supernatants were reduced with 1mM DTT (Life Technologies), and denatured in NuPAGE® LDS Sample Buffer (Life Technologies) at 100°C for 2 minutes. Cell extracts were loaded in equal amounts in 4-12% Bis-Tris Plus Gels (Bolt® Life Technologies) and subjected to electrophoresis at 150V for 1.5 hours. Separated proteins were transferred onto PVDF membranes (Bolt®, Life Technologies) overnight at 60mA. Membranes were probed with antibodies against phosphorylated ERK1/2 diluted 1/1000, phosphorylated FAK Y397 diluted 1/1000, phosphorylated FAK Y576 diluted 1/1000 (all from Molecular Probes), or glyceraldehyde 3-phospho dehydrogenase diluted 1/2000 (GAPDH; Cell Signaling) used to normalize sample input. Primary antibodies were either detected with secondary antibodies (**Supplementary Fig. 1a, b**) or by employing biotinylated secondary antibody sandwich method

(**Supplementary Fig. 1c**) for increased signal strength. All blots were detected by enhanced chemiluminescence using ChemiDoc XRS+ (Biorad). A pre-stained molecular weight marker (SeeBlue®; Life Technologies) was used and imaged with epi-white illumination in ChemiDoc.

Statistics

A total of 1411 acini were analysed across all four experimental conditions for the embedded protocol (EP). An average of 110.3 $\pm$ 31.3 acini per experimental condition were analysed with 3 replicates. A total of 791 acini were analysed across all four experimental conditions for the overlay protocol (OP). An average of 65.9 $\pm$ 19.9 acini per experimental condition were analysed with 3 replicates.

Differences in rheology, roundness, and colony size were tested for statistical significance using a one-sided Mann-Whitney U test. Differences in percent of acini in each category were tested for statistical significance using one-sided student's T-test. To account for multiple two-sample tests within the same data set, we utilized the Bonferroni correction and divided the alpha value by the number of tests conducted. For testing of the percentage of *disrupted*, *invasive*, and *multi-acinar* colonies, $p < 0.0125$ were considered significant. For testing of G' and G'' values of $p < 0.025$ were considered significant. Roundness and area differences were considered significant for a value of $p < 0.05$.

Chimaeric ErbB2 Receptor Dimerizer

MCF10A.ErbB2 cells were obtained from the Muthuswamy laboratory [2]. These cells express a synthetic chimeric transmembrane receptor consisting of a low-affinity growth factor receptor extracellular domain and the cytoplasmic domain of the ErbB2 receptor. The cytoplasmic domain is conjugated to the ligand binding domain of the FK506 binding protein (FKBP), which can be induced to dimerize upon the addition of a synthetic ligand to the culture medium [2]. We

used the B/B dimerizer (Clonetech), also known as AP20187 in place of AP1510 because AP1510 was no longer commercially available. AP20187 was originally synthesized to dimerize Fv domains, which are FKBP proteins bearing a $Phe^{36} \rightarrow Val$ mutation[3]. Although AP20187 binds weakly to FKBP domains, 1µM AP20187 was shown to induce FKBP domain dimerization to a similar extent than that observed with AP1510[3]. We therefore used this 1µM AP20187 concentration to activate dimerization of the chimeric ErbB2 receptor.

MATLAB Acinus Classification Routine

Images were acquired using an Olympus IX81 FluoView 1000 laser scanning confocal microscope. We coded a custom MATLAB (Mathworks) script for classification of acini according to their transmitted light images. The script was designed to blind the user to the experimental conditions the images originated from by randomly calling each image. The graphical user interface (GUI) presents each image to the user who can trace the periphery of each acinus and classify it as *normal*, *disrupted*, *invasive* or *multi-acinar*, visually, using an attached tablet (Wacom Technologies). From each trace, acinus area as well as major axis and minor axis lengths were calculated. Only colonies with areas greater or equal to 490 μm^2 were counted, corresponding to a minimum colony diameter of 25 μm^2. The code then calculates the ratio of major/minor axis length for each *normal* acinus and reclassifies it as d*isrupte*d if the ratio is > 1.3.

References

1 Johnson, K. R., Leight, J. L. & Weaver, V. M. Demistifying the Effects of a Three-Dimensional Microenvironment in Tissue Morphogensis in *Methods Cell Biol.* Vol. Volume 83 (eds Wang Yu-Li & E. Discher Dennis) 547-583 (Academic Press, 2007).

2 Muthuswamy, S. K., Li, D., Lelievre, S., Bissell, M. J. & Brugge, J. S. ErbB2, but not ErbB1, reinitiates proliferation and induces luminal repopulation in epithelial acini. *Nat Cell Biol* **3**, 785-792 (2001).

3 Clackson, T. *et al.* Redesigning an FKBP-ligand interface to generate chemical dimerizers with novel specificity. *Proc. Natl. Acad. Sci. U. S. A.* **95**, 10437-10442 (1998).
